# Searching for success: Development of a combined patient-reported-outcome (“PRO”) criterion for operationalizing success in multi-modal pain therapy

**DOI:** 10.1186/s12913-015-0939-4

**Published:** 2015-07-17

**Authors:** Carolin Donath, Lisa Dorscht, Elmar Graessel, Reinhard Sittl, Christoph Schoen

**Affiliations:** Department of Psychiatry and Psychotherapy, Center for Health Services Research in Medicine, Friedrich-Alexander-Universität Erlangen-Nürnberg, Schwabachanlage 6, 91054 Erlangen, Germany; Interdisciplinary Pain Center, University Clinic Erlangen, Friedrich-Alexander-Universität Erlangen-Nürnberg, Krankenhausstr. 12, 91054 Erlangen, Germany

**Keywords:** Treatment Outcome, PRO, Patient Outcome Assessment, Pain Management, Health Services Research

## Abstract

**Background:**

There is a need for a way to measure success in multi-modal pain therapy that researchers and clinicians can agree upon. According to developments in health services research, operationalizing success should take patient-reported outcomes into account. We will present a success criterion for pain therapy that combines different patient-reported variables and includes validity measures. The usable criterion should be part of a statistically significant and satisfactory model identifying predictors of successful pain therapy.

**Methods:**

Routine data from 375 patients treated with multi-modal pain therapy from 2008 to 2013 were used. The change scores of five constructs were used for the combined success criterion: pain severity, disability due to pain, depressiveness, and physical- and mental-health-related quality of life. According to the literature, an improvement of at least ½ standard deviation was required on at least four of the five constructs to count as successful. A three-step analytical approach including multiple binary logistic regression analysis was chosen to identify the predictors of therapy success with the success criterion as the dependent variable.

**Results:**

A total of 58.1 % of the patients were classified as successful. Convergent and predictive validity data show significant correlations between the criterion and established instruments, while discriminative validity could also be shown. A multiple binary logistic regression analysis confirmed the feasibility; a significant model (Chi^2^ (8) = 52.585; p < .001) that explained 17.6 % of the variance identified the following predictors of therapy success: highest pain severity in the last 4 weeks, disability due to pain, and number of physician visits in the last 6 months.

**Conclusions:**

It is possible to develop a feasible success criterion that combines several variables and includes patient-reported outcomes (“PROs”) with routine data that can be used in a predictor analysis in multi-modal pain therapy. The criterion was based on basic constructs used in pain therapy and used widespread validated self-rating instruments. Thus, it should be easy to transfer this criterion to other institutions.

## Background

Measuring therapy success is one of the most traditional tasks in clinical research. This also applies to the area of pain therapy. The operationalization of successful pain therapy is handled in diverse ways: There are research groups that use pain levels as a measure of success (e.g. [1, 2]), whereas others operationalize success as an increase in social functioning (i.e. ability to engage in productive work), by using days absent due to sickness as an indicator (e.g. [3, 4]), or by using health-care contacts (e.g. [1, 5]). Some studies use patient-centered measures such as quality of life [6]. Until now, there has been no standard procedure or agreement among clinicians who work internationally in pain therapy about which measures to use [7] even though there are national projects that have suggested, for example, that one agreed-upon questionnaire consisting of different instruments be used—for example the German Pain Questionnaire “Deutscher Schmerzfragebogen” [8]. A systematic literature research by Deckert et al. [7] in 2013 identified 71 relevant studies reporting heterogenic outcome measures for multi-modal pain therapy. Most studies used measures from three core health areas (physical, mental, social health), but the number of measures that were used ranged from one measure per study up to 34 outcome parameters in one study. In the 71 publications that were examined, there was not one single outcome criterion that was used in all of the published works [9]. However, the efficacy of multi-modal pain therapy has already been shown, even though there is still a lack of studies on differential indication as reported in the review by van Geen and colleagues [6].

Thus, an agreement on the measurement and operationalization of successful multi-modal pain therapy would be very helpful from a research perspective because this would make comparisons of effects of different pain centers much easier and more reliable both nationally and internationally.

There are two relatively new aspects that should be taken into consideration when developing and suggesting such a measurement tool for wide use:

1. Health Services Research agrees that success (i.e. the “outcome”) of a therapeutic approach should no longer be solely expert-rated [10, 11]. This development is clearly moving in the direction of “patient-reported outcomes” (PROs) that consider the patient’s evaluation of the therapy outcome (e.g. [10, 12]). Such outcomes are seen as a class of study end-points [13] that consider a subjective view on a patient’s satisfaction with the care/treatment that was received [14]. Only if the patient considers the change to be “of worth” will it be considered to be a success. On the other hand, experts might see no improvement in hard parameters, but patients may subjectively feel better, have better pain coping strategies, or feel that their subjective quality of life has improved, etc., and hence rate their therapy outcome as successful. Therefore, a future operationalization of success in multi-modal pain therapy should include patient-reported and patient-rated data. A systematic review showed that well-implemented PROs in routine care improved patient-provider communication and patient satisfaction [15, 16].
2. For reasons of validity and reliability, more than one variable/construct should be used as the criteria for success since each individual construct will not have the same importance for every patient. From a methodological view, such variables should be measured with reliable and objective instruments. The use of already existing instruments that are also used in other areas of clinical research would provide practical help to clinicians internationally. Such instruments should ideally be available in different language versions that have been validated. A large platform on available instruments already exists and is freely accessible for researchers and treatment providers: http://www.proqolid.org/.

### Aims

The first aim of this work is to introduce such a possible measurement of success for multi-modal pain therapy that includes patient-reported data and is comprised of different constructs. The presentation should include empirical evaluation of the criterion.

The second aim is to test the feasibility of the success criterion: Goal is to identify predictors of successful multi-modal pain therapy using the newly developed success criterion as the dependent variable.

The overall goal of having a success criterion in pain therapy and knowing the predictors of success was to direct patients that are suitable and will likely profit of this intensive treatment into the therapy. For clinicians it would be helpful to have a-priori-estimation where they can expect success to be able to use tight resources in the most reasonable way. Furthermore, having a success criterion is a prerequisite for answering further research question such as comparison of different treatment centers or therapists.

## Methods

### Design

The data for the study constitute routine data from patients enrolled in semi in-patient treatment in the interdisciplinary pain center of the University Clinic in Erlangen. The patients participated in a five-week full-time multi-modal pain therapy. The data that we analyzed were collected within the time frame of January 1, 2008 to March 31, 2013. The patients were assessed medically by trained pain therapists (medical doctors and psychologists) with self-reported questionnaires before patients began therapy (“t1”) and after they completed the treatment (“t2”). There were no extra data collected for study reasons. Thus, we developed the success criterion using only data that are already implemented in routine care. The patients were informed about the treatment and the kind of data collection that would accompany their therapy. They gave written consent to be treated and to have their data collected and anonymously analyzed for scientific use. The data were anonymized, and the person who implemented the data analysis did not see any person-related information in the data and also did not see any patients in person so that it would be impossible to form a connection between sensitive data and real persons. The data were treated according to the German and Bavarian legislative rules for data protection. Routine data collection accompanies therapeutic action at the University Clinic Erlangen as a matter of quality assurance. This is in accordance with the ethical commission of the Friedrich-Alexander-Universität Erlangen-Nürnberg.

### Sample

In the time period set aside for this study, a total of 417 patients with a chronic pain condition were enrolled in the 5 week multi-modal pain therapy. Data from 42 patients could not be included in the analysis because of poor data quality—more than 50 % of the assessed variables were missing for those people. Thus, a sample of N = 375 was available for analysis. Single missing data in the N = 375 sample was imputed using the EM-algorithm [17–20].

The sample was comprised of 72.5 % females. The patients had a mean age of 46.9 years (standard deviation: 11.0), 36.3 % were employed/self-employed, 16.5 % were living alone, and 28.3 % had 12 or more years of education.

### Intervention

According to the therapeutic procedures catalogue (“OPS”), multi-modal pain therapy is a multidisciplinary treatment that lasts for at least seven days. It is administered to patients with chronic pain conditions (including tumor pain) and compulsorily involves at least two disciplines.

To be deemed suitable for this kind of treatment (e.g. multi-modal pain therapy), patients are required to have at least three of the following characteristics:

- manifest or impending impairment of quality of life and/or ability to work
- failure of a uni-modal pain therapy in the patient’s anamnestic history or failure of an operative procedure that was administered to relieve pain, or failure of a pain-medication withdrawal treatment
- existing pain medication dependency or abuse
- psychic comorbidity that contributes to manifesting the pain condition
- serious somatic comorbidity

The prerequisites set forth by the German statutory health insurance for multi-modal pain therapy require interdisciplinary diagnostics by at least two disciplines (compulsorily at least psychiatric, psychosomatic, or psychotherapeutic) as well as the parallel application of at least three of the following active therapeutic approaches: psychotherapy, physiotherapy, relaxation methods, occupational therapy, medical training, sensorimotor training, work place training, therapies from the area of art (music, building arts, etc.), or other exercise therapies [21].

Multi-modal pain therapy in Erlangen can be described as a semi-inpatient, intensive, interdisciplinary treatment. The program used in this study was conducted in groups of 8 patients and lasted for 4 to 5 weeks for 6 to 8 hours per day for a total of 5 days per week.

The program included:

- A physical exercise program (every day): general fitness exercises, muscle strengthening exercises, stretching
- Education (every day): anatomy, pain, and physical and mental coping strategies
- Relaxation training (every day): progressive muscle relaxation, applied relaxation
- Optional therapy-related drug treatment or physiotherapy
- Individual physical counseling (once a week): vicious circle of pain, realistic goal determination, individually based medical therapy
- Individual psychological counseling (once a week): cognitive-behavioral intervention, treatment of individual emotional distress

### Development of the success criterion

To come up with the combined success criterion, following considerations were undertaken:

1. The use of valid, wide-spread and consented data in form of patient-rated outcome variables was a prerequisite.
2. At the end a dichotomous variable should result for two reasons: a) facilitating clinical decision making in the future and b) definite classification of success and non-success for prognostic analysis
3. The algorithm resulting in a dichotomous success variable (yes/no) should be as easy as possible to integrate five different constructs.
4. The change patients undergo in therapy should not only be minimally detectable [22], but rather be minimal clinically important.
5. Based on the literature, a distribution-based method that is consented should be used as benchmark for a minimal clinically important difference.

### Five subcriteria

The following instruments were used to construct the combined success criteria for multi-modal pain therapy:

1. Average pain severity in the last 4 weeks: 11-point numeric rating scale of pain severity referring to the “primary pain” ranging from 0 (no pain) to 10 (strongest imaginable pain) – visualized with equal distances between the steps of the scale.
2. PDI: Pain Disability Index [16]—using an equally graded 11-point numeric scale ranging from 0 to 10, this scale measures the degree of impairment in seven different areas of life due to the pain. The mean of the seven areas is used for analysis.
3. ADS: The General (in German: “Allgemeine”) Depression Scale [23] measures dysfunction that can be attributed to depressive symptoms over the last week with 20 items rated on a four-point scale in a self-administered questionnaire. Based on adult norms, a score of 23 points or less suggests average functioning compared with healthy controls, whereas a score of 24 points or more points toward a potentially serious depressive disorder.
4. SF-36: The SF-36 Health Survey [24] was used to operationalize health-related quality of life. It is a patient-rated questionnaire on functional health and well-being with 36 items (see: http://www.sf-36.org/). The physical and mental health summary measures (computed according to the algorithm suggested by the authors) were used.

The variables listed above were chosen because they are widely used both nationally and internationally in pain therapy. To operationalize the constructs of pain severity, disability due to pain, depressiveness, and quality of life, we employed validated and widespread self-report questionnaires to ensure that the measures could be easily transferred to other institutions [8].

#### Integrating the subcriteria into a combined success criterion

Difference values were computed for the five above mentioned constructs. Descriptive statistics for the difference values can be found in Table 1. It was necessary to show an improvement of at least ½ standard deviation (SD) on at least four of the five constructs of the success criterion to count as successful (“Standard Deviation Method”). The difference of ½ standard deviation was used because literature suggests that this is the minimal clinical important difference patients can achieve [25] (independent of statistical significance) and because the threshold of discrimination for changes appears to be approximately that range in different instruments [26]. The chosen method of ½ SD has to subsumed under the distribution-based methods for defining “minimal clinical important differences” [27]. It was a clinical decision to view therapy success as substantial change on most (but not necessarily all), thus 4 out of 5, subcriteria.

**Table 1:** Descriptive statistics for the change values of the five variables incorporated into the combined success criterion

	Minimum	Maximum	Mean	Standard Deviation
Change in average pain severity t2-t1	−7.00	3.11	−1.99	1.94
Change in PDI t2-t1	−8.00	4.57	−1.72	1.89
Change in ADS t2-t1	−46.00	30.00	−12.24	10.77
Change in SF-36 physical t2-t1	−10.86	32.49	6.95	7.08
Change in SF-36 mental t2-t1	−29.61	37.28	8.29	12.24

*t1*: assessment before therapy began

*t2*: assessment after the five-week multi-modal pain therapy was completed

*PDI* Pain Disability Index

*ADS* General Depression Scale

*SF-36* SF-36 Health Survey—Health-related quality of life

While pain severity, disability, and depressiveness should be reduced after therapy, health-related quality of life should be higher (i.e. to show improvement). The construct “depressiveness” was a special case: Here, we looked for improvement only in patients who had been identified as “depressive” at t1. People who had been below the cut-off for depressiveness at t1 were counted as successful as long as they were still below the cut-off after they completed therapy.

The analysis of the five constructs resulted in a dichotomous variable incorporating a decision for each individual: successful—yes/no. Characteristics of the criterion including percentages of patients that are defined as successes are presented in the results section.

### Instruments

In addition to the above-mentioned variables, the following constructs were assessed and used to predict successful pain therapy: age, sex, living situation, school education, employment, number of affected pain regions, localization of the primary pain, affective and sensory pain perception, frequency of primary pain, highest pain severity in the last 4 weeks, lowest pain severity in the last 4 weeks, actual pain severity, subjectively acceptable pain severity after treatment, persuasibility of pain, number of other symptoms accompanying the pain, number of psychic comorbidities, Park-Index*, number of times the personal physician was changed due to failures in pain treatment, number of pain-associated physician visits in the last six months, number of other pain-related treatments (excluding physicians/medical specialists) in the last 6 months, total number of doctors involved because of pain, number of pain-associated hospital visits, number of pain-associated rehabilitation treatments, retirement request pending, current retirement payment, disability request pending, and a work accident as the reason for the pain condition.

*The Park-Index refers to a publication by Park et al. [28] who describe a number of psychic comorbidities that are positively correlated with medically unexplained pain. These comorbidities (Alcohol use disorders, Nicotine Dependence, Mood disorders, Anxiety disorders, Psychotic Disorders, Sleep disturbances) were used in this publication to build a sum score for which a range of 0 to 6 points is possible.

### Statistical analysis

#### Empirical evaluation of the success criterion

To provide an empirical evaluation of the success criterion percentages of patients who fulfilled a certain number of subcriteria (3, 4, 5) are presented. As a sensitivity analysis the required change was set to one SD for each single criterion—theoretically doubling the minimal clinically important difference.

To provide figures for evaluating validity of the success criterion, convergent validity was checked with a) nine scales of the FESV (Questionnaire to assess pain reduction) at the end of treatment; b) with the subjective patient satisfaction at the end of treatment (single item, higher satisfaction was coded with lower values), c) with percentage of patients that reached a subjectively tolerable pain level which they defined at the start of their treatment. The FESV [29] is a self-rated, validated reliable questionnaire measuring therapy success in the scales of “depression reduction”, “fear reduction”, “anger reduction”, “competences in planning”, “cognitive restructuring”, “sense of competence”, “mental distraction”, “countersteering activities”, “relaxation techniques”. For all convergent validity measures correlation and inference statistics with the dichotomous success criterion are presented.

Furthermore predictive validity was analyzed with a) subjective patient satisfaction with treatment one year after discharge and b) with rating of pain severity one year after discharge. For both correlation and inference statistics with the success criterion at the end of treatment are shown.

Discriminative validity was checked by correlation and inference statistics with the MPSS (Chronicity state according to Gerbershagen) rating [16]. This is a measure for chronicity of pain and should by concept not correlate with success.

#### Predictors of successful pain therapy

A three-step analytical approach was chosen to predict successful pain therapy: I) Pre-analysis: Univariate analyses involving binary logistic regressions for 37 potential predictor variables (each alone) and success (dichotomous yes/no) as the dependent variable were carried out. We decided to keep variables for further analysis if they showed a significant (p < .05) or at least a trend toward a significant association (p ≤ .10) with therapy success. II) Multicollinearity: We assessed whether the in Step I significant/trend significant variables were correlated with the other potential predictors. To develop a parsimonious model, we decided to eliminate variables if they had associations with other predictors of r > .500, indicating medium and high degrees of association. The decision about which of two variables had to be eliminated in cases of multicollinearity was based on the degree of association with other variables in the model. The variable that showed higher associations with all other variables was omitted. Thus, the remaining variables were used in the final model. III) Final model: A multiple binary logistic regression analysis was computed with the success criterion as the dependent variable and the predictor variables that were not associated with each other more than r = .500 and that at least showed a trend toward being significantly associated with therapy success in the univariate analysis.

#### Sensitivity analysis predictors

Furthermore, as a sensitivity analysis the variables that had been eliminated in the above described Step I were included in the final model one at a time, thus resulting in analyses with 8 + 1 predictors. This analysis was conducted to account for the very rare possibility that a variable that had a non-significant univariate association with a dependent variable could turn out to have a significant association when it was combined with a certain set of variables. This sensitivity analysis was computed to provide hints about other potentially important variables that might be associated with therapy success.

## Results

### Success criterion

A combined success criterion incorporating the five variables pain severity, pain disability, depressiveness, and health-related quality of life (physical and mental) was developed.

Per definition 58.1 % of patients were counted as successful (at least 4 out of 5 fulfilled criteria). Table 2 compares the frequencies of patients who achieved success on a certain number of the five single criteria. With a broader definition of only 3 out 5 there would be 81 % successful, while a minimal clinical important change in all 5 criteria reached 28.0 %. As a sensitivity analysis Table 2 shows also the number of patients who improved at least 1 Standard Deviation. Using this stricter criterion only 34.2 % would fulfil at least 4 out of 5 criteria. Below 10 % improved at least 1 SD in all 5 subcriteria.

**Table 2:** Frequency of fulfilled success criteria

Number of fulfilled (improved) single success criteria	Combined Success Criterion		Sensitivity Analysis	
	(1/2 SD improvement)		(1 SD improvement)	
	Frequency (N)	%	Frequency (N)	%
0	10	2.7	27	7.2
1	15	4.0	53	14.1
2	46	12.3	83	22.1
3	86	22.9	84	22.4
4	**113**	**30.1**	94	25.1
5	**105**	**28.0**	34	9.1
Total	375	100.0	375	100.0

**Bold**: Definition of successes

**Fig 1:**
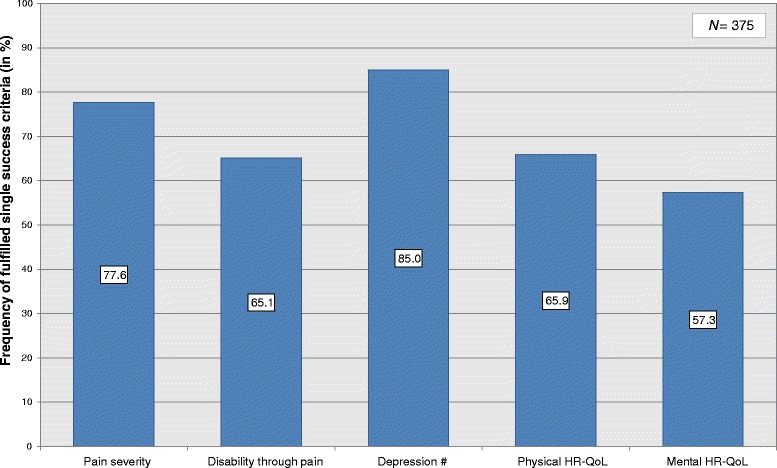
Details of improvement in the constructs of the success criterion. HR-QoL: Health-Related Quality of Life. # Sample size for the depression criterion: N = 223 (only patients who were labelled depressive before therapy began)

The single criterion that had the highest success rate was depressiveness. A total of 223 patients (59.5 %) were above the cut-off before therapy, thus counting as depressive. Of those, 85.0 % improved after therapy (Fig. 1). However, 4 patients (1.1 %) who were not counted as depressive before therapy were above the cut-off after completing therapy and were thus counted as Non-Responders on this single criterion. For health-related quality of life 2/3 or less improved (65.9 % physical, 57.3 % mental).

### Empirical evaluation of the success criterion

#### Convergent validity

The combined success criterion correlates significantly with eight of nine scales of the FESV (Table 3). Except for anger reduction, the patients rated as successful had significant lower values in depression (p < .001), in fear (p = .003), significant higher values in competences in planning (p < .001), in cognitive restructuring (p < .001), in sense of competence (p < .001), in mental distraction (p = .005), in countersteering activities (p = .032) and in relaxation techniques (p = .004) measured with the FESV.

**Table 3:** Empirical evaluation of the success criterion—Convergent validity with the FESV at the end of therapy [29]

	Successes	Non-Successes	T	p	Correlation
	Mean (SD)	Mean (SD)			
FESV scale 1: Depression	44.42 (8.10)	49.65 (9.08)	5.869	<.001	−.291**
FESV scale 2: Fear	44.55 (7.91)	47.35 (9.37)	3.044	<.01	−.160**
FESV scale 3: Anger	48.36 (8.29)	50.08 (8.67)	1.952	n.s.^a^	−.101
FESV scale 4: Competences in planning	61.33 (6.01)	58.90 (6.63)	−3.701	<.001	.188**
FESV scale 5: Cognitive restructuring	58.12 (7.34)	54.31 (8.25)	−4.715	<.001	.237**
FESV scale 6: Sense of competence	56.55 (7.37)	52.98 (8.58)	−4.327	<.001	.219**
FESV scale 7: Mental distraction	57.95 (8.55)	55.38 (8.74)	−2.840	<.01	.145**
FESV scale 8: Countersteering activities	53.50 (8.80)	51.49 (9.11)	−2.155	<.05	.111*
FESV scale 9: Relaxation techniques	60.64 (7.41)	58.17 (8.73)	−2.870	<.01	.151**

* p < .05; ** p < .01; *** p < .001

^a^ p = .052

Therapy success correlates significantly with subjective patient satisfaction at the end of treatment (r = -.261; p < .01). The mean values in satisfaction between successes (M = 1.71) and non-successes (2.13) differ significantly (T = 5.003; p < .001).

Therapy success also correlates significantly with the percentage of patients that reached a subjectively tolerable pain level (Contingence coefficient = .218; p < .01). While of those classified as successful reached 38.5 % their subjective tolerable pain level, in the group of non-successes reached this only 17.8 % (Chi^2^ = 18.666; p < .001)

#### Predictive validity

Success according to the presented criterion correlates significantly with subjective patient satisfaction with treatment one year after discharge (r = -.272; p < .01). Successful patients have a significantly higher rated satisfaction (displayed in lower values because of the scale) (M = 1.88 vs. M = 2.34; T = 3.611; p < .001) one year after finished treatment.

Furthermore a significant correlation between pain severity one year after discharge and the definition as a succeed is evident (r = -.193; p < .05). Thus patients defined as successful have significantly lower pain severity one year after discharge (M = 6.9) than patients defined as not successful (M = 7.7) (T = 2.654; p = .009).

#### Discriminative validity

The Gerbershagen rating describing the chronicity of pain is neither with the sum score (r = .046) nor with the stadium (r = .056) significantly or clinical relevantly correlated with therapy success. Successes and non-successes do not differ in their mean sum score significantly (M = 8.91 vs. M = 8.76; T = -.822; p = .412).

### Predictors of success in multi-modal pain therapy

Step 1 – Pre-Analysis: The binary-logistic regression analyses revealed the following 9 significant predictors of success(successes coded 1, non-successes coded 0): affective pain perception (p = .016; OR: 1.03), average pain severity in the last 4 weeks (p < .001; OR: 1.34), highest pain severity in the last 4 weeks (p < .001; OR: 1.43), lowest pain severity in the last 4 weeks (p = .005; OR: 1.13), actual pain severity (p = .003; OR: 1.14), disability through pain (PDI) (p < .001; OR: 1.42), depressiveness (ADS) (p = .004; OR: 1.03), mental-health-related quality of life (SF-36) (p = .006; OR: 0.98), and number of pain-associated physician visits in the last six months (p = .056; OR: 0.98),. Furthermore, the two variables age (p = .097; OR: 0.98) and school education (p = .077; OR: 1.50) were significant at an alpha-level of .10. Detailed results for each of the 37 regression analyses can be found in Table 4. As a result, 11 variables were kept and used in the multicollinearity analysis.

**Table 4:** Pre-Analysis (Step 1)—Univariate associations with success in multi-modal pain therapy (binary logistic regressions)

	Regression Coefficient β	Standard error	Wald	df	p	OR	95 % Confidence Interval for OR	
							Lower Value	Upper Value
Age	−.016	.010	2.755	1	**.097**	0.984	0.966	1.003
Sex	−.229	.237	0.933	1	.334	0.795	0.500	1.266
Living situation	−.161	.280	0.331	1	.565	0.851	0.492	1.473
School education	.408	.231	3.122	1	**.077**	1.504	0.956	2.365
Employment	.145	.217	0.444	1	.505	1.156	0.755	1.769
Number of affected pain regions	.001	.014	0.011	1	.918	1.001	0.974	1.029
Localization of primary pain:								
head	.180	.289	0.387	1	.534	1.197	0.679	2.111
upper body	−.286	.217	1.738	1	.187	0.751	0.491	1.149
lower body	−.103	.234	0.194	1	.660	0.902	0.571	1.427
joints	.571	.377	2.288	1	.130	1.769	0.845	3.706
whole body	.063	.350	0.032	1	.858	1.065	0.536	2.113
Pain perception affective (SES)	.028	.012	5.842	1	**.016**	1.029	1.005	1.053
Pain perception sensory (SES)	.001	.015	0.006	1	.936	1.001	0.972	1.031
Frequency of primary pain	−.192	.238	0.655	1	.418	0.825	0.518	1.314
Average pain severity in the last 4 weeks	.289	.063	20.695	1	**<.001**	1.335	1.179	1.511
Highest pain severity in the last 4 weeks	.359	.083	18.456	1	**<.001**	1.431	1.215	1.686
Lowest pain severity in the last 4 weeks	.126	.045	7.828	1	**.005**	1.134	1.038	1.239
Actual pain severity	.133	.045	8.830	1	**.003**	1.143	1.046	1.248
Acceptable pain severity after treatment	.023	.062	0.137	1	.712	1.023	0.906	1.156
Persuasibility of pain	.276	.252	1.200	1	.273	1.318	0.804	2.160
Number of symptoms accompanying pain	.002	.047	0.003	1	.959	1.002	0.914	1.099
Number of psychic comorbidities	.142	.150	0.898	1	.343	1.153	0.859	1.547
Park-Index^a^	.273	.190	2.068	1	.150	1.314	0.906	1.905
Disability due to Pain (PDI)	.348	.066	28.218	1	**<.001**	1.417	1.246	1.611
Depressiveness (ADS)	.029	.010	8.387	1	**.004**	1.029	1.009	1.049
Physical-health-related quality of life (SF-36)	−.021	.014	2.095	1	.148	0.980	0.953	1.007
Mental-health-related quality of life (SF-36)	−.025	.009	7.413	1	**.006**	0.976	0.959	0.993
Number of personal physician switches	.060	.056	1.162	1	.281	1.062	0.952	1.184
Number of pain-associated physician visits in the last six months	−.023	.012	3.657	1	**.056**	0.977	0.954	1.001
Number of pain-related other treatments in the last 6 months	−.003	.007	0.200	1	.655	0.997	0.982	1.011
Number of doctors involved because of pain	−.010	.025	0.151	1	.698	0.990	0.943	1.040
Number of pain-associated hospital visits	−.026	.056	0.222	1	.638	0.974	0.873	1.087
Number of pain-associated rehabilitations	.027	.071	0.146	1	.702	1.027	0.894	1.180
Retirement request pending	−.008	.595	0.000	1	.989	0.992	0.309	3.183
Current retirement payment	−.108	.292	0.138	1	.710	0.897	0.507	1.589
Disability request pending	.292	.216	1.821	1	.177	1.339	0.876	2.047
Work accident as reason for pain condition	−.083	.422	0.038	1	.845	0.921	0.402	2.107

^a^consists of Alcohol use disorders, Nicotine Dependence, Mood disorders, Anxiety disorders, Psychotic Disorders, Sleep disturbances: sum index with range of 0 to 6

**Bold:** significant at a level of p < .05 or at a level of p ≤ .10

Step 2 – Multicollinearity Analysis: the following variables were eliminated from the final model: depressiveness (r = -.764 with mental-health-related quality of life), average pain severity in the last 4 weeks (r > .500 with highest, lowest, and actual pain severity), and actual pain severity in the last 4 weeks (r > .500 with highest, lowest, and average pain severity). Thus, 8 variables remained in the final modal.

Step 3 – Full model—multiple binary logistic regression analysis: A significant model (Chi^2^ (8) = 52.585; p < .001) that explained 17.6 % of the variance identified a total of three variables predicting success (successes coded 1, non-successes coded 0) in multi-modal pain-therapy: disability due to pain (p < .001; OR: 1.36), highest pain severity in the last 4 weeks (p = .025; OR: 1.26), and number of pain-associated physician visits (p = .008; OR: 0.97). Patients who were more impaired and had a smaller number of pain-associated physician visits in the last 6 months (in addition to the multi-modal pain therapy) had a higher probability of success. The detailed results for the 8 predictors that were included are shown in Table 5.

**Table 5:** Full model (Step 3)—Multiple binary logistic regression analysis with combined success criterion as the dependent variable

	Regression Coefficient β	Standard-error	Wald	df	p	OR	% Confidence Interval for OR	
							Lower Value	Upper Value
Age	−.020	.011	3.481	1	.062	0.981	0.960	1.001
School education	.430	.262	2.695	1	.101	1.537	0.920	2.569
Pain perception affective (SES)	−.011	.015	0.499	1	.480	0.990	0.961	1.019
Lowest pain severity in the last 4 weeks	−.029	.057	0.250	1	.617	0.972	0.868	1.088
Highest pain severity in the last 4 weeks	.229	.102	5.013	1	**.025**	1.257	1.029	1.535
Disability due to Pain (PDI)	.306	.077	15.803	1	**<.001**	1.358	1.168	1.580
Mental-health-related quality of life (SF-36)	−.017	.010	2.746	1	.098	0.983	0.964	1.003
Number of pain-associated physician visits in the last six months	−.036	.014	6.947	1	**.008**	0.965	0.939	0.991

**Bold:** significant at a level of p < .05

### Sensitivity analyses

A sensitivity analysis explored whether the variables that had been excluded in Step 1 could have been significant predictors of success when included one by one in the full model with the 8 chosen variables. As a result, the 26 additional variables did not change the results of the 8 chosen variables. The three significant predictors (pain severity, disability due to pain, and physician visits) remained significant in all cases. The variable age was a further significant predictor in the models with the additional predictors “number of pain-associated hospital visits” (age: p = .047) and “number of symptoms accompanying the pain” (age: p = .048). All other chosen variables remained unchanged in their associations. Only one additional predictor had a significant association with success when included in the full multivariate model: “disability request pending”. It was associated such that success in therapy was more probable when there was no disability request pending (p = .008).

## Discussion

### Success criterion

This work originated in the practical routine care of chronic pain patients. After every block of multi-modal pain therapy in the pain therapy center, therapists claimed that they “somehow knew” who had benefitted and who had not—however, this was more of a gut instinct rather than a measurable effect. Thus, a need to be able to operationalize success in multi-modal pain therapy was evident. A further goal was to optimize patient recruitment and allocation to an expensive specialized treatment such as semi-inpatient multi-modal pain therapy. It was important to know who was benefitting from such therapy—but in order to answer that question; agreement about what was meant by “benefitting” was needed first.

Thus, first, there was agreement that success in multi-modal pain therapy needed to be measured multi-modally and thus with different constructs. Second, the measurement should be implemented with constructs that had been assessed as valid and relevant for the patient; therefore, patient reported outcomes were utilized. Third, the measurement should be feasible, easy to implement within routine care (or already implemented), valid, reliable, and objective; therefore, we used well-known measures with tested quality criteria that were widely used (at least in Germany). Fourth, literature-based knowledge on minimally clinical important difference should be incorporated.

As a consequence, we developed a combined success criterion including five single criteria (pain severity, disability due to pain, depressiveness, and quality of life—physical and mental). We defined success as improvements of at least ½ standard deviation on at least four of the five single criteria.

A first empirical evaluation showed evident convergent validity, predictive and discriminant validity. Patients defined as successes had more favourable values than patients defined as non-successes in the instruments used for convergent and predictive validity. The strength of the correlations seems to be low. However, this can be explained by the concept: correlation of a multi-domain-construct with a single criterion. Thus, the combined success criterion is to heterogenic to show high correlation with for example pain severity.

### Limitations

A critical point is that the success criterion does not include the social aspect of re-establishing the ability to become gainfully employed or to work, which has been used as a single success criterion in many international studies (e.g. [3, 4]). However, this reflects the special situation in Germany where the pressure of regaining the ability to work might not be as high because the financial coverage is rather long and sufficient. The therapeutic experience in multi-modal pain therapy often shows the opposite effect: if the therapist expresses the expectation that the patient should to be able to work again, the patient might react with a pain “relapse” and a worsening of symptoms if the patient believes he/she has “a right to be sick.” Therefore, the ability to be gainfully employed should be used as a success criterion if it is expressed by the patient (in the sense of “PRO”) and only for such patients—but not as a general success criterion for all.

Reflecting the results concerning therapy success, it has to be stated that immediately after therapy completion, more than half of the patients improved markedly on at least four of the five constructs. However, to judge the effectiveness of multi-modal pain therapy, this result is insufficient and needs to be validated with follow-up data. This is a limitation at the moment. We plan to do this as soon as the routine data are available, and the results will be published consequently. However, the mean amount of change in the single criteria is comparable to results reported in the literature with pain patients treated with several diverse treatments concerning the mental health related quality of life [30, 31], physical health related quality of life [30, 32], pain severity [33] and functioning [34].

A further limitation is, besides that it concerns in this manuscript data of a single center, that we were bound to the assessment instruments implemented in routine pain care. The advantage of this on the other hand is that those instruments are used in all multimodal pain therapy centers in Germany because of the dissemination of the German Pain Questionnaire [8] and thus transferability of the criterion was easily given.

With the decision for ½ standard deviation as minimal clinically important difference (MCID) there might be a certain amount of change that is undetected since the MCID is not equivalent with the minimally detectable change [22]. For the latter we would have needed to count even 1-point-changes in each of the five measures. This has methodological problems though, since a) the scaling of the five instruments is not equivalent and b) the rate of measurement errors/differences in measurement by chance seen as clinical relevant improvement would have afflicted validity of the success criterion.

The presented combined success criterion has to be seen as a first (pilot) version. Adaption of the criterion concerning number and kind of included single criteria might be necessary depending on the results of further examination: a) in other treatment centers, b) with follow-up data c) when routine assessment instruments are changed.

Up to now our approach of measuring success does not reflect individual patient preferences and/or goals. It would also be an adaptation possibility to define success in the single criteria more dependent on the change goals of the patient himself.

In conclusion, we developed a feasible, combined, PRO-success criterion for multimodal pain therapy. However, the challenge will be to raise awareness in practical working pain therapy centers that there is a need for such a criterion and a need for comparable outcome measures. Even though the pain research community frequently asks “that a standard outcome be used across pain studies” [35] and “for more systematic assessment” [36], work by Deckert and colleagues has shown a great deal of diversity in how success is measured in multi-modal pain therapy [7, 9]. Thus, the ongoing multi-center, federally funded, VAPAIN-project will hopefully raise awareness of this gap [37].

#### Predictors of therapy success

Concerning the predictors, we found that the patients with especially heavy burdens profited the most. Patients with more severe pain when therapy began and with larger impairments in their daily life functioning due to their pain were associated with a higher probability of therapy success. There could be two reasons for these findings. One is a methodological one: People with high scores on the pain severity and pain impairment scales had better chances of improving substantially than patients who began with low scores (ceiling effect). The second reason is a motivational one: pain therapists frequently report that patients who are seriously impaired and bothered by their dysfunction have the highest levels of motivation to change their lives. To a large degree, multi-modal pain therapy is about changing habits. Changing habits is not easy and is often inconvenient. Thus, a patient must be motivated (see Transtheoretical Model according to Prochaska & DiClemente [38]) to bridge the intention-action gap. From Motivational Interviewing according to Miller & Rollnick [39], it is known that the will to change has to come from the patient himself/herself, and therapists can only support patients by bringing up the topic of change. Such motivation seems to be more prevalent in patients who are enduring a great deal of suffering from their pain condition and whose secondary gain from illness is rather low. There is support from the literature that the readiness to self-manage pain is an important predictor of the completion [40] of and the functional outcome [41] of a treatment program.

Like our study, a study from the Netherlands also found that high scores on disability were associated with recovery five and 12 months after therapy in patients with chronic nonspecific low back pain [42]. A German study of fibromyalgia patients also confirmed this association: higher impairment at baseline was predictive of a clinically significant decrease in impairment after treatment; greater physical impairment prior to treatment increased the likelihood of having a substantial decrease in functional impairment due to pain after therapy was completed by about 4.24 times [43]. Furthermore, another study of chronic pain patients reported a better prognosis for patients with a higher baseline pain score just as we found [44]. On the other hand, the “pain score” (in the sense of higher values implying higher pain severity) seems to be associated with an unsuccessful return to work in patients with low back pain after an inpatient hospital intervention [45]. However, we need to ask whether “the ability to be gainfully employed” should be used as a single outcome.

The third significant predictor of success after completing therapy in our study was the number of pain-related physician visits in the last six months. Success was higher for patients with a smaller number of doctors visits. The interpretation could be that highly impaired pain patients had stopped visiting doctors for out-patient care because they had decided that such visits were not helping them. So perhaps they were focusing on this planned intensive semi-inpatient 5-week multi-modal treatment and had put a lot of their hopes in that specific therapy and were therefore highly motivated to succeed. One early study also reported results in this direction: especially non-depressed patients with chronic pain seemed to profit from treatment if they had fewer previous types of treatment [46]. In another study [44], a worse prognosis was reported for chronic pain patients who had had more treatment in the past.

There is a frequently recycled discussion in pain therapy about whether patients who are in the process of applying for disability pension should be included in multi-modal pain therapy. The hypothesis is that those patients will not/cannot improve because of their conflict of interest. There are studies from Denmark supporting this hypothesis [47, 48]. However, our results from the main analysis do not confirm this hypothesis. Neither a pending retirement request nor a pending disability request was univariately significantly associated with therapy success. Thus, excluding such patients in the first place does not appear to be justified. However, we found in a sensitivity analysis that therapy success may be more probable when there is no disability claim pending.

In our study, younger age showed a trend toward being associated with a higher probability of therapy success. This direction of association has also been reported in other studies (e.g. [42, 44]).

Interestingly, the Patient-Reported Outcome (PRO) that we focused on in this study with regard to the success of multi-modal pain therapy still seems to be different from therapy satisfaction as one Australian study had suggested [49].

In conclusion, there are not many published works that have focused on the predictors of success in multi-modal pain therapy. The works cited in our discussion make reference to other pain management strategies, unimodal approaches, or rather unspecific pain therapy concepts. As Morasco and colleagues stated in their review: “Limited data are available to identify predictors of treatment outcome” [50].

## Conclusions

From the perspectives of both researchers and clinicians, a combined “PRO” criterion for assessing success in multi-modal pain therapy is desirable. There are two reasons for this: 1) it models the multifactorial genesis and therapy of chronic pain better than one single category criterion, and 2) a decision about whether a patient is considered a success or not is necessary for almost all analyses that would follow such as differences in outcomes between different patient collectives, observation of long-term outcomes of applied health services, etc. The combined success criterion that we developed involves well-established constructs whose measurement is implemented in routine care in pain treatment centers. A first empirical evaluation confirms evidence of content validity.
